# Brain Structural and Functional Alterations in Mice Prenatally Exposed to LPS Are Only Partially Rescued by Anti-Inflammatory Treatment

**DOI:** 10.3390/brainsci10090620

**Published:** 2020-09-07

**Authors:** Francesca Aria, Sara A. Bonini, Valentina Cattaneo, Marika Premoli, Andrea Mastinu, Giuseppina Maccarinelli, Maurizio Memo

**Affiliations:** Department of Molecular and Translational Medicine, University of Brescia, Viale Europa 11, 25123 Brescia, Italy; f.aria@unibs.it (F.A.); v.cattaneo@studenti.unibs.it (V.C.); m.premoli002@unibs.it (M.P.); andrea.mastinu@unibs.it (A.M.); giuseppina.maccarinelli@unibs.it (G.M.); maurizio.memo@unibs.it (M.M.)

**Keywords:** maternal immune activation, lipopolysaccharide, anti-inflammatory drugs, cortical structure, behavior, neurodevelopmental disorders

## Abstract

Aberrant immune activity during neurodevelopment could participate in the generation of neurological dysfunctions characteristic of several neurodevelopmental disorders (NDDs). Numerous epidemiological studies have shown a link between maternal infections and NDDs risk; animal models of maternal immune activation (MIA) have confirmed this association. Activation of maternal immune system during pregnancy induces behavioral and functional alterations in offspring but the biological mechanisms at the basis of these effects are still poorly understood. In this study, we investigated the effects of prenatal lipopolysaccharide (LPS) exposure in peripheral and central inflammation, cortical cytoarchitecture and behavior of offspring (LPS-mice). LPS-mice reported a significant increase in interleukin-1β (IL-1β) serum level, glial fibrillary acidic protein (GFAP)- and ionized calcium-binding adapter molecule 1 (Iba1)-positive cells in the cortex. Furthermore, cytoarchitecture analysis in specific brain areas, showed aberrant alterations in minicolumns’ organization in LPS-mice adult brain. In addition, we demonstrated that LPS-mice presented behavioral alterations throughout life. In order to better understand biological mechanisms whereby LPS induced these alterations, dams were treated with meloxicam. We demonstrated for the first time that exposure to LPS throughout pregnancy induces structural permanent alterations in offspring brain. LPS-mice also present severe behavioral impairments. Preventive treatment with meloxicam reduced inflammation in offspring but did not rescue them from structural and behavioral alterations.

## 1. Introduction

Neurodevelopmental disorders (NDDs) are a group of disorders associated with impaired brain development. Developmental brain dysfunctions may first appear in the postnatal period, such as in autism spectrum disorders (ASD) and Down syndrome, or in adulthood as in neuropsychiatric disorders, with emotional, relational and cognitive deficits [1,2,3]. Development of the nervous system is extremely complex and tightly regulated by a series of mechanisms, such as cells proliferation, migration and differentiation, consequently an alteration in one or more of these mechanisms could lead to NDDs [4,5,6]. Notoriously, the origins of NDDs are so varied and heterogeneous that finding a preventive cure for these disorders is currently an extremely demanding challenge.

In our study, we focused our attention on an emerging potential cause of NDDs: maternal immune activation (MIA). In the last 20 years, several epidemiological studies have shown an association between MIA and NDDs [7,8,9,10,11,12,13,14]. MIA is based on the concept that the activation of maternal immune system can induce brain alterations during fetal development [15,16,17,18]. The complex and various roles that immune molecules and glia cells play in our body could explain this. The common idea for immune molecules and glia cells is that they are involved during an inflammatory response (peripheral and central inflammation) to protect the body from harmful pathogens [19,20,21]. Interestingly, recent research has shown that the immune molecules and glia cells could be also involved in the regulation of different neurodevelopmental processes, such as neuronal plasticity and function [22,23,24,25]. This revolutionary idea supports the hypothesis of MIA as a possible pathogenic mechanism for NDDs [15,26,27,28].

In the past years, the MIA animal models showed that a maternal infection could alter brain development inducing functional and behavioral alterations [19,29,30,31,32,33,34,35,36,37]. Now, the prominent question is which biological mechanisms are changing due to inflammation in the offspring’s brain. To date, it is known that a pro-inflammatory stimulus induces alterations in the maternal immune system and in the placental microenvironment resulting in a possible priming of NDDs [16,38]. These issues, together with the difficulty of using drug therapy during pregnancy, have focused the attention on the possibility of curing symptoms in the offspring instead of preventing alterations in brain development.

To generate a MIA mouse model, a pro-inflammatory stimulus, lipopolysaccharide (LPS), a component of Gram-negative bacteria, was used. LPS was chosen for its easier handling and lower variability when compared to other pathogens used in MIA models [38,39]. LPS is a typical stimulus used to mimic infection in many animal studies of MIA; indeed, it induces a well-characterized inflammatory response by activating toll-like receptor 4 (TLR4) [40,41,42] and a consequent increase in several pro-inflammatory factors, such as cytokines, and glia cells activation [32,42,43,44]. It has also been reported to cause typical NDDs symptoms in the offspring, such as behavioral deficits, both social and cognitive [45,46].

The aim of this work was to better understand the biological mechanisms underlying the inflammatory response induced by LPS in the offspring and possibly reveal a potential new method in the treatment of NDDs. We used meloxicam, a well-known nonsteroidal anti-inflammatory drug (NSAID) cyclooxygenase-2 (Cox-2) preferential throughout pregnancy. Notoriously, there are two isoforms of Cox: Cox-1, constitutive isoform [47] and Cox-2, inducible isoform [48]. Although there are contrasting opinions concerning the use of NSAIDs during pregnancy, several studies reported that only non-selective Cox inhibitors or selective Cox-1 inhibitors induce teratogen effects during pregnancy. Moreover, collateral side effects were observed at high doses of NSAIDs and rarely at low doses [49,50,51,52,53]. Since Cox-2 is the main enzyme responsible for inflammatory response during an infection [54,55] and meloxicam (MLX) has a good safety index at low doses during pregnancy, it was chosen as anti-inflammatory treatment for dam mice.

In this study, we investigated the offspring for: (i) the effects of a chronic pro-inflammatory stimulus during the pre-natal period; (ii) the effects of a concomitant treatment with an anti-inflammatory drug, in order to establish the potential protective effect of an anti-inflammatory drug in a MIA model. We evaluated the effects that LPS and/or meloxicam have on both central and peripheral inflammation, cortical cytoarchitecture and behavior.

## 2. Materials and Methods

### 2.1. Animals

In this study, wild type (B6;129PF2) mice were used. Mice were purchased from The Jackson Laboratories (Bar Harbor, ME, USA). Animals were housed two-to-three per cage in a 12-h light/dark cycle (light phase: from 8:00 a.m. to 8:00 p.m.), with food and water available ad libitum. The cage size was 15 cm wide × 35 cm long × 12 cm deep. Temperature (22 °C) and humidity (50% ± 1) in the cage were automatically regulated by Sealsafe Aero System by individually ventilated cages with EPA filters (Tecniplast Group, Varese, Italy). Experiments were conducted in conformity with the European Communities Council Directive of 1986 (86/609/EEC) and approved by the Italian Ministry of Health (910/2016-PR), Animal Care and Use Committee of the University of Brescia.

### 2.2. MIA Model

Female mice were mated with male mice (1 female −1 male) at 12–14 weeks of age for a period of 24 h. The morning after mating, the presence of a vaginal plug was verified, and in case of positivity, this was considered the gestational day (GD) 0. Successively, female mice (from now on called dams) were divided randomly in 3 groups of 4–6 dams each.

The first group of dams was treated with vehicle (VH, phosphate-buffered saline, PBS) subcutaneously (s.c.) on alternate days from GD1 to the end of pregnancy. The second group of dams was injected with 0.3 mg/kg of LPS from *Escherichia coli* (L-3129 serotype 0127:B8, MilliporeSigma, Merck KGaA, Darmstadt, Germany) subcutaneously (s.c.) on alternate days [56] from GD1 to the end of pregnancy. The third group of dams was treated with LPS and MLX simultaneously. This last group will be further described in detail below.

### 2.3. Drug Treatment

MLX (MilliporeSigma, Merck KGaA, Darmstadt, Germany), a well-known preferential Cox-2 inhibitor, was administered (2 mg/kg per day) by oral gavage throughout pregnancy to dams, 30 min before LPS injection. LPS (0.3 mg/kg) was injected subcutaneously (s.c.) on alternate days from GD1 until the end of pregnancy. The dosage of MLX (2 mg/kg per day) was chosen based on median effective dose of previous studies [57,58,59].

### 2.4. Offspring

Offspring obtained from the three groups of dams were divided in three groups (VH-mice, LPS-mice and LPS + MLX mice) and housed two to three per cage after the weaning (4 weeks). All the structural and behavioral analysis carried out in this study were conducted on the offspring at different time periods: pups (ultrasonic vocalizations and homing test), juvenile mice (olfactory habituation/dishabituation test) and adult mice (open filed test, reciprocal social interaction test, marble burying test, home cage observation test). Behavioral tests on pups and juvenile mice were carried out on both genders, whereas tests on adult mice were done only on male mice. After the behavioral tests, mice were sacrificed either to obtain serum for ELISA testing, or brains for immunohistochemistry and minicolumn analysis.

### 2.5. Immunohistochemistry

Three adult male mice per group were used for immunohistochemistry (IHC). At the end of behavioral experiments, mice were anesthetized with chloral hydrate (400 mg/kg) and perfused with 4% paraformaldehyde (PFA). Then, brains were fixed, cryoprotected and coronally crysectioned at 20 µm. IHC for glial fibrillary acidic protein (GFAP), marker for astrocytes, and ionized calcium-binding adapter molecule 1 (Iba1), marker for microglia, was performed. The following primary antibodies were used: α-Iba1 rabbit (W.D. 1:500, Wako Chemicals GmbH, Neuss, Germany), α-GFAP mouse (W.D. 1:300, Sigma Aldrich, Saint Louis, MO, USA). The following secondary antibodies were used: biotinylated α-rabbit (W.D. 1:1000, Vector, Burlingame, CA, USA) and biotinylated α-mouse (W.D. 1:1000, Vector, Burlingame, CA, USA), respectively. Immunochemistry signal was detected with 3,3-diaminobenzidine tetrahydrochloride (DAB). For each animal, a complete series of one-in-eight sections (160 μm apart) through the cortex was analyzed, and positive cells were counted. An average of 8 –10 sections per animal was used. Images of the cortex were acquired by using a ×20 (Iba1) and a ×40 (GFAP) objectives with an Olympus BX41 microscope (Olympus Life Science, Waltham, MA, USA). Iba1- and GFAP-positive cells were counted in the entire cortex for each group. For the cell count, we drew squares with a defined area (width of 200 µm and height of 600 µm for Iba1 cells, and width of 300 µm and height of 400 µm for GFAP cells) by using the ImageJ software (National Institute of Health, Bethesda, MD, USA). A minimum of 6 areas for each section were counted. Analyses have been performed in a blinded manner. Data are expressed as Iba1- and GFAP-positive cells per area.

### 2.6. Cortical Column Analysis

Three adult male mice for group were used for cortical column analysis. Coronal brain sections were stained with cresyl violet (Nissl staining). Images of Nissl-stained brain sections were acquired by using a ×10 objective of an Olympus BX41 microscope. Successively, cortical minicolumns were semiautomatically analyzed using the ImageJ software as previously described [60]. Anterior cingulate (AC) cortex (corresponding to bregma 1.70 and 1.94 mm) and somatosensory (SS) cortex (corresponding to bregma −1.28 and −1.64 mm) were analyzed in both cerebral hemispheres of three mice per group. A mouse brain atlas (Franklin and Paxinos) was used as a reference to ensure the correct positioning of the cortical images acquired. A rectangular area with the size of a minicolumn (region of interest, ROI) (width 60 µm; height 400 µm) was marked in layers II–IV, where columnar organization of the cortex is clearly defined. Up to 15 ROIs per section, placed at regular, non-overlapping intervals across the image were analyzed. Average cell density and total path length ratio (TPLR), an index of column vertical organization, were measured in each ROI. Only cells having a size of 70–350 µm^2^ (pyramidal neurons) were included in the count. The cell area range defined was set to restrict cell count to pyramidal neurons (excluding glia and interneurons) and to avoid multiple cell clusters. Analyses have been performed in a blinded manner.

### 2.7. ELISA Test

Levels of interleukin-1β (IL-1β) were quantified in serum of 6 VH, 5 LPS and 5 LPS + MLX adult male mice. Mouse IL-1β ELISA kit (Invitrogen, ThermoFisher Scientific, Vienna, Austria) was used and the test was performed following the manufacturer’s protocol.

### 2.8. Behavioral Testing

Behavioral tests were carried out during the light phase of circadian cycle, between 9:00 a.m. and 5:00 p.m. In addition, 32–38 pups, 20 juvenile mice and at least 7 adult male mice (4–6-month-old) per group were used. During the tests, the operator remained in an adjacent room, separated with a dark sliding door from the test room. All the behavioral analyses have been performed in a blinded manner.

### 2.9. Pups Behavior

#### 2.9.1. Ultrasonic Vocalizations in Pups

Ultrasonic Vocalizations (USVs) emitted by pups after maternal separation for 3 min on post-natal day (PND) 4, 6, 8, 10 and 12 were analyzed using Avisoft Bioacoustics system as previously described [61,62]. Briefly, USVs were recorded with ultrasound microphone (UltraSoundGate CM16, Avisoft Bioacoustics, Glienicke/Nordbahn, Germany) sensitive to frequencies of 10–180 kHz and analyzed with SasLab Pro (version 5.2; Avisoft Bioacoustics, Glienicke/Nordbahn, Germany). Number and duration of calls were analyzed.

#### 2.9.2. Homing Test

Homing test has been performed as previously described [63]. Briefly, on PND 14, pups were transferred in a plexiglas T-maze (arm size: 10 cm length × 8 cm width). The maze was divided into a start arm, clean arm (covered by clean sawdust) and nest arm (covered by sawdust collected from the nest of the pup cage). The nest and clean arms were alternated between right and left between each pup. Each pup was placed in the start arm and was free to explore the maze for 5 min. Videos captured by a camera above the maze were analyzed by the operator, measuring first latency to reach the nest, time spent in the nest and in the clean arms and locomotor activity by square crossing.

### 2.10. Juvenile Mice Behavior

#### Olfactory Habituation/Dishabituation Test

Olfactory habituation/dishabituation test has been performed as previously described [62,64]. Briefly, juvenile mice (6 weeks old) were placed in an empty cage and exposed to different odors, classified as non-social (vanilla and orange) and social odors (urine from same-gender or opposite-gender mice), in three consecutive trials of 2 min duration each. Time of olfactory investigation (considered as sniffing activity up to 2 cm distance from applicator) of each mouse was evaluated.

### 2.11. Adult Mice Behavior

#### 2.11.1. Open Field Test

Open field test was performed as previously described [65]. Briefly, each mouse was acclimated in the procedure room for 10 min, then placed in the center of the arena (40 × 40 cm Plexiglas box) and allowed to freely explore for 5 min, during which time the mouse was recorded by a camera placed on top of the arena. The Plexiglas box was cleaned after each test session to delete any odor deposited by previous mice. Mouse locomotor activity, total distance travelled, and average speed were automatically analyzed with Ethovision XT software (version 13, Noldus, Wageningen, The Netherlands).

#### 2.11.2. Reciprocal Social Interaction Test

Reciprocal social interaction test was performed following the Silverman protocol [66]. Briefly, mice were individually housed for 48 h preceding behavioral testing. For the test, after a period of acclimation of 10 min in the procedure room, each mouse was exposed to an age, sex and strain-matched unfamiliar subject (intruder subject) for 10 min. The test was video recorded and analyzed with Observer XT (version 14.1, Noldus, Wageningen, The Netherlands). Specific social (nose-to-nose sniffing, anogenital sniffing, body sniffing, following, wrestling, mounting, body contact) and non-social (cage exploring, self-grooming) behaviors were evaluated.

#### 2.11.3. Home Cage Observation Test

In the home cage observation test mice were individually placed in a clean cage, with water and food, and observed for 1 h. The test was video recorded by a digital camera (GigE monochrome, Noldus, Wageningen, Netherlands) and analyzed by two independent observers. Time spent on self-grooming activity was measured.

#### 2.11.4. Marble Burying Test

For this test, a protocol modified from Kedia [67] was applied. In detail, a rat cage (37 length × 18 width × 21 cm height) was filled with a 5-cm-deep sawdust layer. In the habituation phase, each mouse was placed in the cage and allowed to explore freely for 10 min after which it was removed. Twelve dark glass marbles were spaced evenly in a 3 × 4 grid on the surface of the sawdust. The mouse was then placed in the cage and was allowed to explore and interact with the marbles for 5 min. At the end of the test, the mouse was removed, and the number of the marbles buried were counted (a marble was considered buried if at least two-thirds of it was covered with sawdust). The test was recorded with digital camera (Noldus, Wageningen, The Netherlands) and analyzed by the operator.

### 2.12. Statistical Analysis

Statistical analysis was performed by Prism 6 software (GraphPad, San Diego, CA, USA). The data were analyzed by one- or two-way analysis of variance (ANOVA) followed by post hoc tests. For body weight, ultrasonic vocalizations pups analysis and the olfactory habituation/dishabituation tests, the repeated measurements (RM) Two-way ANOVA, followed by Tukey’s multiple comparison test was used. For homing test, the RM Two-way ANOVA, followed by Sidak’s multiple comparisons test was performed. For serum analysis, counts of GFAP and Iba1 cells, open field testing, as well as social and repetitive tests, the One-way ANOVA followed by the Dunnett multiple comparison test was used. For analysis of minicolumns, the One-way ANOVA followed by a Tukey multiple comparison test was used. Data were presented as the means ± standard error mean (S.E.M.), with the statistical significance level set at *p* < 0.05.

## 3. Results

### 3.1. LPS Induced Both Peripheral and Central Inflammation, Partially Prevented by Meloxicam

**Figure 1:**
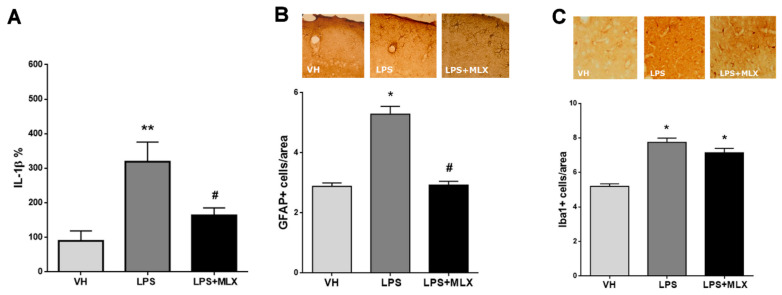
Effects of maternal exposure to lipopolysaccharide (LPS) on peripheral and central inflammation on adult offspring. (**A**) Effect of LPS on interleukin-1β (IL-1β) serum levels in adult offspring (F (2, 13) = 9.60, *p* = 0.0028). ** *p* = 0.0022 for LPS-mice vs. vehicle (VH)-mice. # *p* < 0.037 for LPS + meloxicam (MLX) mice vs. LPS-mice. *N* = 6 VH, 5 LPS, 5 LPS + MLX. (**B**) In the inset: representative images of glial fibrillary acidic protein (GFAP) staining in VH-, LPS- and LPS + MLX-mice cortex. In the graph: effect of LPS on GFAP-positive cells as marker of astrocytes in the cortex of adult offspring (F (2, 709) = 61.02, *p* < 0.0001). * *p* < 0.0001 for LPS- vs. VH-mice. # *p* < 0.0001 for LPS + MLX-mice vs. LPS-mice. *N* = 3 per group. (**C**) In the inset: representative images of ionized calcium-binding adapter molecule 1 (Iba1) staining in VH-, LPS- and LPS + MLX-mice cortex. In the graph: effect of LPS on Iba1-positive cells as marker of microglia in the cortex of adult offspring (F (2, 1569) = 46.37, *p* < 0.0001). * *p* < 0.0001 for LPS-, LPS + MLX-mice vs. VH-mice. *N* = 3 per group. Data were analyzed by one-way ANOVA followed by Tukey’s post-test analysis.

The first milestone of this study was to verify whether chronically injected LPS in the mothers during pregnancy actually generated a MIA model by measuring inflammatory markers in adult offspring. Activation of the maternal immune system during pregnancy has been previously shown to induce long-lasting effects on immune system in the offspring [16,33,68]. In particular, we measured in adult mice offspring (from now on called LPS-mice): (a) IL-1β levels in the serum as a marker of peripheral inflammation by ELISA; (b) Iba1- and GFAP-positive cells in the cortex by IHC as markers of central inflammation. As reported in Figure 1A, LPS induced a significant increase in IL-1β serum levels compared to VH-mice and the MLX treatment was able to prevent it (% mean ± S.E.M.: VH 89.74 ± 28.91, LPS 319.1 ± 56.87, LPS + MLX 163 ± 21.76). This indicates that LPS treatment during pregnancy induced a long-lasting peripheral inflammation in the offspring, and this effect was blocked by the MLX treatment. Looking at the brain, LPS-mice reported a significant increase in both Iba1- and GFAP-positive cells in the cortex compared to VH-mice; MLX was able to prevent only the increase in GFAP-positive cells, but not the increase in Iba1-positive cells (GFAP+ cells/area mean ± S.E.M.: VH 2.89 ± 0.12, LPS 5.29 ± 0.26, LPS + MLX 2.93 ± 0.12; Iba1+ cells/area mean ± S.E.M.: VH 5.2 ± 0.14, LPS 7.75 ± 0.24, LPS + MLX 7.14 ± 0.26) (Figure 1B,C). GFAP is a marker of astrocytes, whereas Iba1 is a microglial marker. Taken together, these results suggest that a chronic LPS exposure during pregnancy alters the offspring’s immune system in the long term and that MLX can partially rescue neuroinflammation in LPS-mice.

### 3.2. Abnormal Organization in the Cortex of Adult LPS-Mice

**Figure 2:**
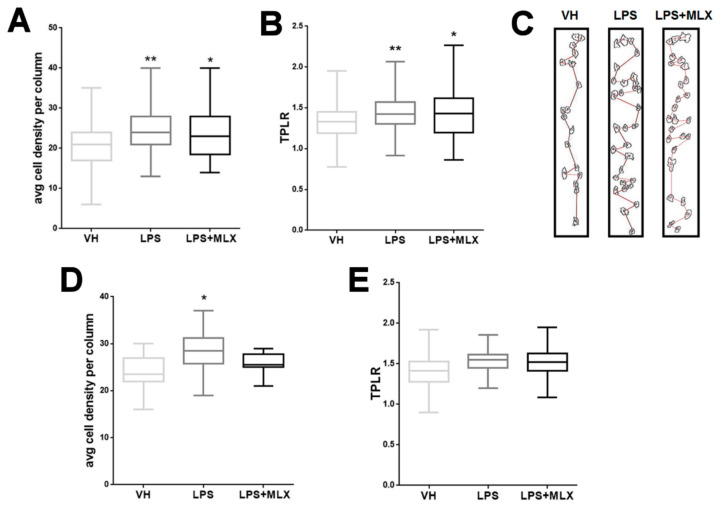
Abnormal columnar organization in the cortex of adult LPS-mice. Graphic representation of data obtained from the minicolumn analysis in somatosensory (SS) cortex (**A**,**B**) and anterior cingulate (AC) cortex (**D**,**E**) of adult LPS-mice. (**A**) Average cell density per column in the SS cortex (F (2, 597) = 29.33, *p* < 0.0001). ** *p* < 0.0001 for LPS-mice vs. VH-mice. * *p* = 0.0003 for LPS + MLX-mice vs. VH-mice. (**B**) Path length ratio (TPLR) in the SS cortex (F (2, 604) = 21.57, *p* < 0.0001). ** *p* < 0.0001 for LPS-mice vs. VH-mice. * *p* = 0.0003 for LPS + MLX-mice vs. VH-mice. (**D**) Average cell density per column in AC (F (2, 75) = 10.27, *p* = 0.0001). * *p* < 0.0001 for LPS-mice vs. VH-mice. (**E**) No significant difference among groups was observed in the TPLR of AC. *N* = 3 per group. Data were analyzed by one-way ANOVA followed by Tukey’s post-test analysis. (**C**) Representative images of minicolumns in VH-, LPS- and LPS + MLX-mice SS cortex.

To examine the effect of a maternal LPS treatment on the cytoarchitecture of offspring, columnar organization in the somatosensory (SS) cortex and anterior cingulate (AC) cortex of three mice per group was analyzed. These two areas were chosen for their importance in the contest of neurodevelopmental disorders [30,69,70,71]. Disruption of columnar organization can be detected by different parameters, such as an altered number of cells and cell positioning in the column [72]. Only cells with an area of 70–350 µm^2^ (pyramidal neurons) were included in the analysis. LPS-mice showed an increase in cell density within a defined column in both the SS and AC cortex compared with VH-mice; MLX treatment did not significantly modify this parameter (cell density per column mean ± S.E.M.: somatosensory cortex, VH 21.05 ± 0.30, LPS 24.27 ± 0.29, LPS + MLX 23.85 ± 0.79; anterior cingulate cortex, VH 23.77 ± 0.52, LPS 28.23 ± 1.05, LPS + MLX 25.83 ± 0.65) (Figure 2A–C). Furthermore, the path length ratio (TPLR), a measurement of linearity of the cell column, was increased in LPS-mice SS cortex compared with VH-mice and also in this case no significant difference was observed after the MLX treatment (TPLR mean ± S.E.M.: VH 1.32 ± 0.012, LPS 1.44 ± 0.011, LPS + MLX 1.44 ± 0.037) (Figure 2B–D). No significant differences in TPLR were reported in the AC cortex between the groups (TPLR mean ± S.E.M.: VH 1.42 ± 0.036, LPS 1.53 ± 0.036, LPS + MLX 1.52 ± 0.06). These data demonstrate, for the first time, that LPS prenatal exposure causes specific cortical structure alterations in the offspring, that cannot be prevented by the MLX treatment.

### 3.3. Effect of Prenatal LPS Exposure on Pups

**Figure 3:**
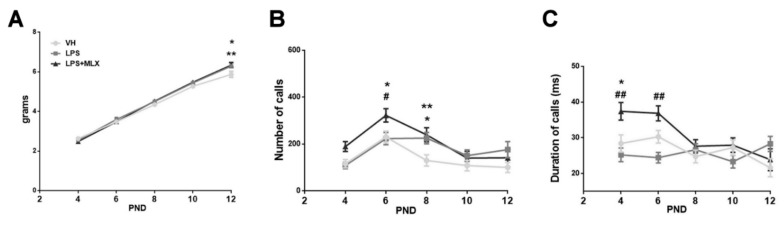
Effects of maternal exposure to LPS on pups’ development and vocalization. *N* = 38 VH, 38 LPS, 32 LPS + MLX. (**A**) Body weight was analyzed in pups on post-natal day (PND) 4, 6, 8, 10 and 12 (F interaction (8, 510) = 1927, *p* = 0.05). * *p* = 0.0016 for LPS-mice vs. VH-mice on PND 12. ** *p* = 0.0005 for LPS + MLX-mice vs. VH-mice on PND 12. Data were analyzed by 2-way ANOVA followed by Dunnett’s post-test analysis. (**B**) Number of calls emitted on PND 4, 6, 8, 10, 12 by pups in response to maternal separation during three minute session (F interaction (8, 416) = 2325, *p* = 0.018). * *p* = 0.03 for LPS + MLX-mice vs. VH-mice and # *p* = 0.02 for LPS + MLX-mice vs. LPS-mice on PND 6. * *p* = 0.02 for LPS-mice vs. VH-mice on PND 8. ** *p* = 0.01 for LPS + MLX-mice vs. VH-mice on PND 8. (**C**) Duration of calls emitted on PND 4, 6, 8, 10, 12 by pups in response to maternal separation during a three-minute session (F interaction (8, 416) = 4781, *p* < 0.0001). * *p* = 0.008 for LPS + MLX-mice vs. VH-mice on PND 4. ## *p* = 0.0002 for LPS + MLX-mice vs. LPS-mice on PND 4. ## *p* = 0.0001 for LPS + MLX-mice vs. LPS-mice on PND 6. Data were analyzed by repeated measurements (RM) Two-Way ANOVA followed by Tukey’s post-test analysis.

Body weight was measured in pups until PND 12; no significant differences emerged between groups, except for the last day (Figure 3A). Indeed, at day 12 both LPS- and LPS + MLX-pups reported a significant increase in body weight compared with VH pups (weight in grams, mean ± S.E.M.: VH 5.91 ± 0.15, LPS 6.4 ± 0.10, LPS + MLX 6.35 ± 0.13). This body weight increase has already been reported previously in the literature in mice exposed to LPS [73,74].

During this time lapse, pups’ vocalizations were studied during maternal separation. As reported in Figure 3B, the USVs-emitted curve of VH-pups has the common U-inverted shape, with a pick at day 6. The LPS treatment induced a significant increase in USVs number at day 8 compared to VH-pups emitting USVs (number of calls, mean ± S.E.M.: VH 130.1 ± 24.04, LPS 225.3 ± 24.21). Interestingly, LPS + MLX-pups emitted significantly more USVs than LPS-pups (number of calls, mean ± S.E.M. at day 6: LPS 223.4 ± 26.5, MLX + LPS 322.4 ± 28.45). Additionally, looking at calls duration, meloxicam treatment induced a significant increase in duration of calls compared to VH (duration of calls (milliseconds) mean ± S.E.M. at day 4: VH 28.43 ± 2.4, LPS + MLX 37.41 ± 2.46) and to LPS-pups (duration of calls (milliseconds) mean ± S.E.M. at day 4: LPS 25.23 ± 1.9, LPS + MLX 37.41 ± 2.46; at day 6: LPS 24.45 ± 1.47, LPS + MLX 36.87 ± 2.10) (Figure 3C). No gender differences were found (Figure S1). For the first time, these data report that a chronic LPS injection during pregnancy causes communication alteration in pups, and that a maternal meloxicam treatment exacerbates this abnormal vocalization pattern.

### 3.4. Prenatal LPS Exposure Does Not Affect Social Recognition and Motility in Pups

**Figure 4:**
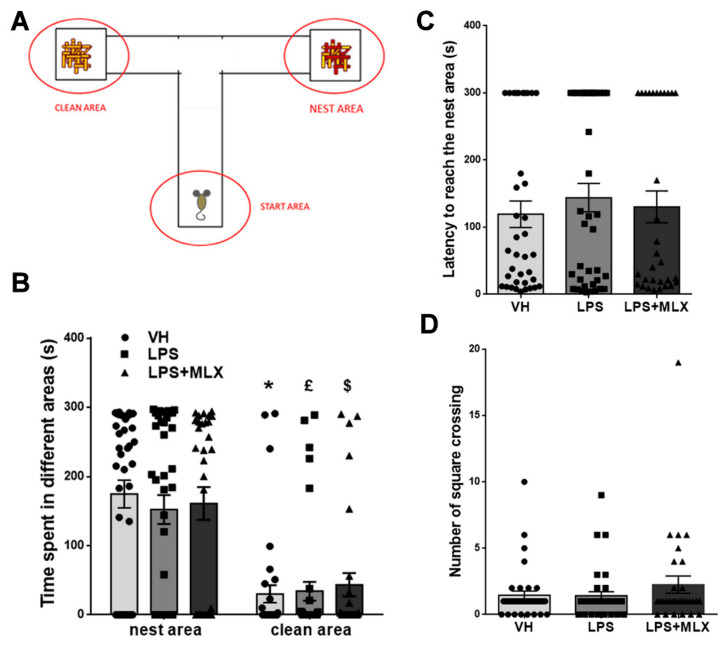
Effect of prenatal LPS exposure on social recognition in pups. *N* = 38 VH, 38 LPS, 32 LPS + MLX. (**A**) Schematic representation of homing test. (**B**) Social recognition was evaluated in homing test on PND 14 by measuring the time (s) spent in nest area and clean area (F_zone_ (1, 103) = 50.25, *p* < 0.0001). * *p* < 0.0001 for time spent in nest area vs. clean area for VH-mice. £ *p* < 0.0005 for time spent in nest area vs. clean area for LPS-mice. $ *p* < 0.001 for time spent in nest area vs. clean area for LPS + MLX-mice. Data were analyzed by Two-Way ANOVA followed by Sidak’s post-test analysis. No significant difference among groups was observed in latency to reach the nest area (**C**) and number of square crossings (**D**).

Social recognition in pups was evaluated by means of the homing test; this test permits measurement of the ability of pups to discriminate between their nest (litter-sawdust) odors and new ones (clean sawdust and start empty area) (Figure 4A). In addition, their motor ability was evaluated. At first, the time spent in each of the two arms of the apparatus was measured: no difference among groups was observed (Figure 4B), they all spent significantly more time in the nest area (seconds spent in the nest area, mean ± S.E.M.: VH 174.7 ± 20, LPS 152.16 ± 20.92, LPS + MLX 161 ± 23.70; seconds spent in the clean area, mean ± S.E.M.: VH 30.19 ± 12.6, LPS 34.16 ± 13.68, LPS + MLX 43.61 ± 16.88). Additionally, the latency to reach the nest area presented no significant differences between groups (latency in seconds to reach the nest area, mean ± S.E.M.: VH 119.4 ± 19.76, LPS 144 ± 21.24, LPS + MLX 130.4 ± 23.72) (Figure 4C). Concerning locomotor activity, no difference emerged among groups, in terms of square crossing (number of square crossing mean ± S.E.M.: VH 174.7, LPS 1.42 ± 0.30, LPS + MLX 2.26 ± 0.65) (Figure 4D). No gender differences were found (Figure S2). These data demonstrate that maternal LPS injection during pregnancy did not change social recognition and motility in pups and that the MLX treatment did not affect them.

### 3.5. Olfactory Deficit in Juvenile LPS-Mice

**Figure 5:**
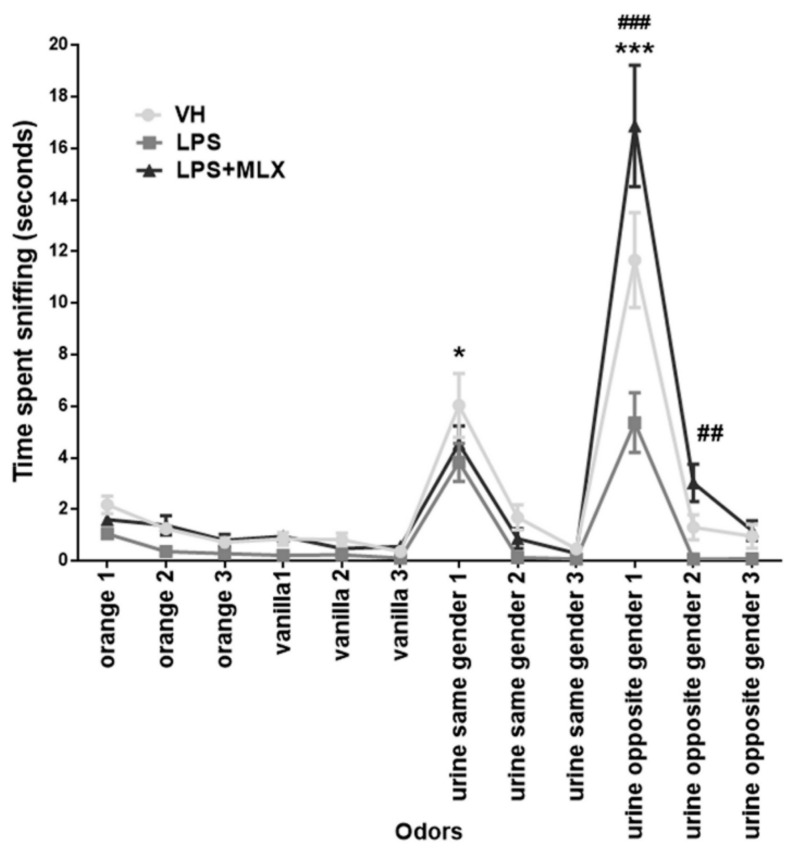
Graphic representation of data collected during olfactory habituation/dishabituation test by measuring the time in seconds (s) spent by adolescent mice in sniffing cotton-tipped swabs saturated with different odors (F_interaction_ (22, 627) = 6.8, *p* < 0.0001). * *p* = 0.04 for urine the same gender 1, LPS-mice vs. VH-mice. *** *p* < 0.0001 for urine opposite gender 1, LPS-mice vs. VH-mice and LPS + MLX-mice vs. VH-mice. ### *p* < 0.0001 for urine opposite gender 1, LPS + MLX-mice vs. LPS-mice. ## *p* = 0.003 for urine opposite gender 2, LPS + MLX-mice vs. LPS-mice. Data were analyzed by Two-Way ANOVA followed by Tukey’s post-test analysis. *N* = 20 VH, 20 LPS, 20 LPS + MLX.

Mice use sense of smell for a wide range of behaviors, such as learning and sociality. To study the effects of prenatal LPS stimulus on olfaction in the offspring, we tested juvenile mice with the olfactory habituation/dishabituation test. During the test, different odors (social and non-social) are introduced to the mouse three times. As expected, VH-mice were not interested in non-social odors (orange and vanilla), but highly interested by social odors (urine from same-gender or opposite-gender mice) (Figure 5). Interestingly, LPS-mice showed significant reduction of interest in social odors compared to VH-mice (time sniffing in seconds, mean ± S.E.M.: urine same gender 1, VH 6.03 ± 1.24, LPS 3.83 ± 0.74; urine opposite gender 1, VH 11.66 ± 1.84, LPS 5.36 ± 1.16). Surprisingly, meloxicam was able to partially restore interest in social odors in mice. Indeed, LPS + MLX-mice spent significantly more time sniffing urine of opposite-gender mice compared to both LPS- and also VH-mice (time sniffing in seconds, mean ± S.E.M.: urine opposite gender 1, VH 11.66 ± 1.84, LPS 5.36 ± 1.16, LPS + MLX 16.87 ± 2.36; urine opposite gender 2, VH 1.3 ± 0.49, LPS 0.06 ± 0.05, LPS + MLX 3.02 ± 0.72). No significant effects were observed with urine of same-gender mice. No gender differences were found (Figure S3). These data suggest that juvenile mice prenatally treated with LPS present a deficit in social odors and that MLX treatment partially prevents it.

### 3.6. Effect of Prenatal LPS Exposure on Adult Mice Behavior

**Figure 6:**
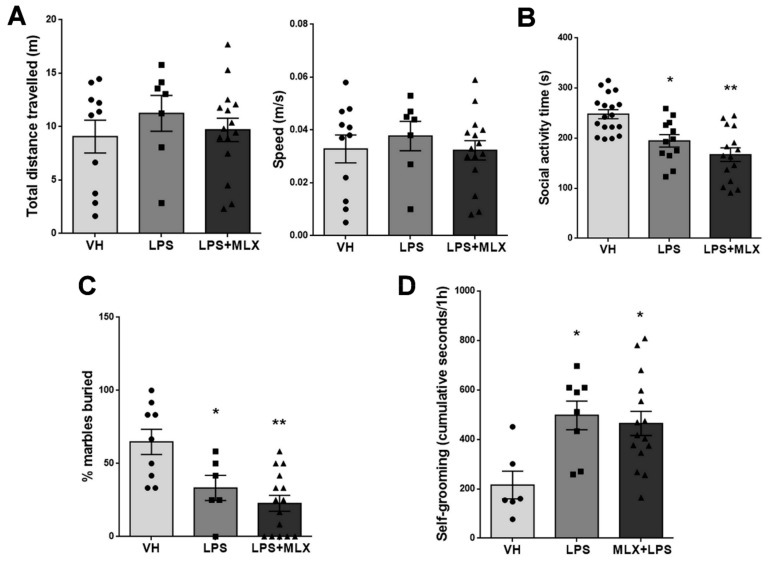
Effect of prenatal LPS exposure on adult offspring behavior. (**A**) Graphic representation of data collected in the open field test by automatically measuring the total distance travelled and the speed. No significant difference among groups was observed. *N* = 10 VH, 7 LPS, 15 LPS + MLX. (**B**) Time spent by adult offspring engaging in social activity during male–male social interaction test (F (2, 42) = 14,04, *p* < 0.0001). * *p* = 0.007 for LPS-mice vs. VH-mice. ** *p* < 0.0001 for LPS + MLX-mice vs. VH-mice. Data were analyzed by One-Way ANOVA followed by Tukey’s post-test analysis. *N* = 18 VH, 12 LPS, 15 LPS + MLX. (**C**) Graphic representation of data collected in the marble burying test by manually measuring the number of marbles buried (F (2, 27) = 9857, *p* = 0.0006). * *p* = 0.02 for LPS-mice vs. VH-mice. ** *p* = 0.0003 for LPS + MLX-mice vs. VH-mice. *N* = 9 VH, 7 LPS, 15 LPS + MLX. (**D**) Time spent by adult offspring on self-grooming during home cage observation test (F (2, 26) = 5487, *p* = 0.01). * *p* = 0.01 for LPS-mice vs. VH-mice and LPS + MLX-mice vs. VH-mice. Data were analyzed by One-Way ANOVA followed by Dunnett’s post-test analysis. *N* = 7 VH, 8 LPS, 15 LPS + MLX.

Locomotion and exploratory activity of adult VH-, LPS- and LPS + MLX-mice were measured in the open field test. No significant differences in total distance travelled and speed were observed between groups (total distance travelled (meters) mean ± S.E.M.: VH 9.08 ± 1.54, LPS 11.25 ± 1.68, LPS + MLX 9.7 ± 1.1; speed (m/s) mean ± S.E.M.: VH 0.033 ± 0.005, LPS 0.04 ± 0.005, LPS + MLX 0.03 0.003) (Figure 6A).

Male–male reciprocal social interaction testing was performed to analyze social behavior. During the 10-min test, the time spent engaging in social and non-social activities was quantified for the analysis (for a detailed quantification of time spent for each single activity see Figure S4). Prenatal LPS exposure significantly reduced social interaction in mice compared with VH-mice; MLX treatment did not significantly modify this behavioral deficit (time (seconds) spent engaging in social activity, mean ± S.E.M.: VH 247.7 ± 9.01, LPS 247.7 ± 9.01, LPS + MLX 167 ± 13.54) (Figure 6B).

The marble-burying test was also performed in order to check an innate and spontaneous behavior in mice: the tendency of burying objects in the sawdust. The number of marbles buried was analyzed at the end of the 5-min test. Both LPS- and LPS + MLX-mice showed a significant reduction in number of marbles buried compared with VH-mice (% marbles buried mean ± S.E.M.: VH 64.81 ± 8.65, LPS 33.33 ± 8.6, LPS + MLX 22.78 ± 5.42) (Figure 6C).

Furthermore, self-grooming activity, a common stereotyped behavior, was analyzed in mice with the home cage observation test. LPS-mice showed a significant increase in time spent on self-grooming compared with VH-mice, and again MLX could not modify this negative behavior (time (seconds) spent on self-grooming: VH 22.78 ± 5.42, LPS 497 ± 57.7, LPS + MLX 464.2 ± 48.71) (Figure 6D). Altogether, these data on mice behavior indicate that prenatal LPS exposure provokes significant behavioral deficits in the offspring, such as a reduction of social behavior and digging activity, other than an increase in stereotyped behavior. Meloxicam treatment has no protective effects on such behavioral alterations.

## 4. Discussion

In this study, LPS, the major component of the outer membrane of Gram-negative bacteria, was used as pro-inflammatory stimulus to generate a murine MIA model. Commonly, in MIA models the inflammatory stimulus is administrated in a specific pregnancy window to study how inflammation affects a specific stage of embryonic development [9,75,76,77,78]. Here, we decided to mimic an exposure to low doses of an inflammatory agent throughout pregnancy and to study how this exposure can influence brain development. LPS was chosen for its easier handling and lower variability compared to other pathogens used in MIA models. Successively, inflammation, cortical cytoarchitecture and behavior in the offspring were analyzed.

At first, inflammation level in the offspring was measured in order to detect potential long-lasting effects on immune system deriving from a prenatal LPS exposure. Adult LPS-mice reported a significant increase in IL-1β serum levels, with IL-1 β one of the major pro-inflammatory markers. Furthermore, an increase in Iba1- and GFAP-positive cells, markers of neuroinflammation, was observed in the cortex of adult LPS mice. These results are in agreement with other MIA studies that showed that maternal immune activation could induce alterations in microglia and astrocytes activity [56,79,80,81,82,83]. On the contrary, other studies on MIA models did not observe glial cells activation [84,85,86]. This variability among studies could be explained by the diversity of protocols used to induce a MIA model in rodents, such as different immune stimulants, doses and different gestational time points. In order to study a possible preventive pharmacological treatment, meloxicam, a preferential Cox-2 inhibitor, was administered chronically to dams during pregnancy. Meloxicam showed a low toxicity during pregnancy compared to other NSAIDs [46,48]. Actually, meloxicam treatment resulted to be compatible with mice pregnancy; indeed, no toxic or teratogen effects were reported [45,46,47,48,49]. Pharmacological treatment on dams with meloxicam was able to prevent the LPS-dependent increase in IL-1β levels in the serum of the offspring. However, only the increase in GFAP-positive cells, but not of Iba1-positive cells, was significantly prevented by meloxicam treatment in the offspring brain cortex. Several studies in the literature report the protective effect of the treatment with anti-inflammatory drugs on MIA models, but most of them have been performed on the offspring [31,84,85,86,87]. Only a few studies have been conducted on dams, but not using pharmacological treatment with anti-inflammatory drugs [88,89,90,91].

Successively, we investigated whether prenatal LPS stimulus induced permanent structural alteration in the offspring’s brain. With this aim, we performed a minicolumnar cortical structure analysis. In the field of neuropsychiatry, the idea that alteration of minicolumnar cortical structure and function (minicolumnopathy) may be responsible for aberrant brain function is growing [66,70,92]. Minicolumns are basal and functional units of the brain cortex and minicolumnopathies are modifications of minicolumnar homeostasis induced by various factors, such as neuroinflammation, gene expression or environmental factors, typically present in several NDDs [89,90,91,92]. We demonstrated, for the first time, that LPS prenatal exposure induces aberrant brain cytoarchitecture. In particular, we found a disorganization of minicolumnar structure in both somatosensory (SS) and anterior cingulate (AC) cortex. We focused our attention on these specific cortical areas due to their involvement in altered processes typical of NDDs [69,71,93,94]. LPS-mice reported an increase in cell density in both SS and AC cortex and an increase in TPLR, an index of column organization, in the SS cortex. The meloxicam treatment was unable to prevent structural abnormalities observed in LPS-mice. Other functional and structural changes in the brain of MIA offspring have been reported in several studies. For instance, magnetic resonance imaging (MRI) studies have identified enlarged ventricles and volume reductions of several brain regions in adult rodent and non-human primate MIA offspring [79,80,81,82,95]. Another study found no change in total brain volume, but unilateral variation in the relative volume of specific brain regions [96]. Moreover, other studies have shown alteration in neuronal density in specific brain areas such as the dentate gyrus of the hippocampus and the subiculum of fetal MIA offspring or in the cerebellum, hippocampus and cortex of adult MIA offspring [30,95,97,98,99,100,101,102,103]. A study performed on a MIA model induced at late gestation has reported only a slight increase in the density of neurons in the corpus callosum and an increase in the density of somatostatin interneurons [104]. These data are in accordance with functional studies in a MIA offspring’s brain, in which are reported alterations in the migration of pyramidal neurons (expressed as reduction in the production of reelin) both in the neonatal [30], developing [17,105] and adult [106] brain of MIA offspring.

In order to evaluate behavioral effects of MIA, offspring were tested during infancy, youth and adulthood.

At first, development parameters, ultrasonic vocalizations (USVs) and behavior in pups were analyzed. In the first two weeks of life (PNDs 4–14), pups born after a chronic maternal LPS injection showed USVs alterations. USVs are social signals of communication and represent a relevant aspect to be analyzed in animal models of neurodevelopmental disorders [107,108,109]. We found a typical inverted U-shaped emission pattern with a peak in PND 6 in VH-pups. This pattern was altered in LPS-pups, which presented an increase in number of calls at PND 8. Meloxicam treatment preserved the typical U-shaped USV pattern, if it did not prevent the increase in number of calls emitted. The results observed for LPS-mice are in agreement with several other studies, reporting that mice exposed to MIA present alterations in USVs. In particular, some studies reported a decrease in the number or duration of USVs [88,110,111,112,113], while others found an increase in the number of USVs [114,115]. These differences probably depend to the species (rat or mouse) and to the genetic strain used, different pro-inflammatory stimuli, timing of immune insult and pups’ age.

Furthermore, during PNDs 4, 6, 8, 10 and 12, pups’ body weights were measured. A significant increase in body weight in LPS- and LPS + MLX- versus VH-pups was observed only on PND 12. This weight increase may depend on metabolic consequences due to LPS exposure, as previously reported [73,74].

Successively, social recognition and motor ability were evaluated in the homing test. LPS or meloxicam did not affect any of these parameters: VH-, LPS- and MLX+LPS-pups spent more time in the nest area compared to the clean area, whereas they did not show differences in number of square crossing and latency to reach the nest area (motor ability). Instead, juvenile offspring showed significant differences in olfactory habituation/dishabituation test. Notably, LPS-mice presented a significant interest reduction in social odors that was partially prevented by meloxicam. This effect may be explained with motivational impairments observed after prenatal exposure to LPS [116].

Finally, behavioral analyses were performed in adult mice. Reciprocal social interaction testing revealed a significant reduction in social activity in LPS-mice compared to VH-mice. Moreover, a significant increase in stereotyped and repetitive behavior was observed in adult LPS-mice. These results are in agreement with several other studies, where murine MIA models reported a deficit in social behavior, such as social affiliation [88,112,116] or interaction [113,117,118], repetitive and stereotyped behavior [88,112,119,120,121].

Taken together, these results demonstrated that a chronic prenatal LPS exposure causes alterations in the cytoarchitecture of specific cortical areas, with impairments in social and repetitive behaviors. Moreover, a preventive pharmacological treatment with meloxicam in dams, despite being able to counteract inflammation, did not rescue from structural and behavioral alterations LPS-mice. This could be explained by the complexity of inflammatory response induced by LPS after its interaction with TLR-4 in the maternal system [16,29,30,45,122]. Indeed, after this interaction, LPS induces activation of cytokines, chemokines, prostaglandins, leukotrienes and hypothalamic–pituitary–adrenal axis, provoking a systemic inflammatory response in dams [16,42,123,124,125,126,127]. This response affects fetal development by inducing activation of pro-inflammatory factors, such as IL-1, IL-6 and Tumor Necrosis Factor-α (TNF-α) [16,32,128]. All these factors can influence brain development of the offspring in an unknown way, causing all the typical alterations of MIA models.

## 5. Conclusions

In conclusion, in this work we demonstrated that chronic prenatal exposure to an inflammatory stimulus (LPS) led to long-lasting activation of the immune system in the offspring. Furthermore, LPS prenatal exposure induced structural alterations in specific cortical areas and behavioral impairments in the offspring, particularly in social and stereotyped behavior. Preventive treatment with meloxicam reduced inflammation in the offspring, as observed both with peripheral and central markers, but did not rescue from structural and behavioral deficits. This is the first study reporting the effects of a chronic pharmacological treatment with an anti-inflammatory drug performed during pregnancy. Only a few studies have focused on a prenatal intervention on mothers to mitigate the maternal immune activation and protect the offspring in the critical phase of gestational neurodevelopment. In particular, to prevent some behavioral deficits observed in MIA offspring, dams were treated with probiotics [119], specific anti-cytokine antibodies [88,89], and environmental enrichments [129,130], or again with maternal dietary supplementation with zinc [131], n-3 polyunsaturated fatty acids [132,133] or N-acetyl-cysteine (NAC) [134]. A pharmacological treatment on dams was performed by Cui and colleagues. In this study, they showed that ibuprofen (a non-selective NSAID) treatment on dams at the time exposure to LPS, was unable to block the alteration in hippocampal neurons [39].

The majority of pharmacological treatments in MIA models have been performed over different postnatal periods of the offspring. For instance, minocycline, a microglia modulator, prevented the emergence of MIA-induced behaviors and changes in pro-inflammatory cytokines in the adult brain [31,84,87]. A study conducted in juvenile rats prenatally treated with poly I:C showed that a treatment with a selective inhibitor for Cox-2, celecoxib, protected adult rats from developing some behavioral deficits [135]. Finally, several postnatal dietary intervention studies reported promising data. For instance, treating juvenile MIA offspring with a ketogenic diet or glucoraphanin was able to rescue offspring from some behavioral deficits [100,136].

Finally, during neuronal development, a multitude of factors are involved in correct neuronal proliferation, migration, positioning and contact formation. It is probable that the meloxicam-dependent cyclooxygenase inhibition is insufficient to prevent all the NDDs-related triggering mechanisms induced by LPS. Further studies are needed to better understand the complex connection between inflammation, brain architecture and brain function.

## Supplementary Materials

The following are available online at https://www.mdpi.com/2076-3425/10/9/620/s1, Figure S1: Effects of maternal exposure to LPS on male and female pups’ vocalization, Figure S2: Effect of prenatal LPS exposure on social recognition in male and female pups, Figure S3: Graphic representation of data collected during olfactory habituation/dishabituation test by measuring the time in seconds (s) spent by adolescent male (A) and female (B) mice in sniffing cotton-tipped swabs saturated with different odors, Figure S4: Detailed representation of time spent doing social activities such as sniffing, following, mounting, contact, wrestling and non-social activities as self grooming and exploring.
