# Supplementary figures and images for: Brain Structural and Functional Alterations in Mice Prenatally Exposed to LPS Are Only Partially Rescued by Anti-Inflammatory Treatment
Source: Brain Sci. 2020 Sep 7;10(9):620. doi: 10.3390/brainsci10090620 (PMC7564777; doi:10.3390/brainsci10090620)

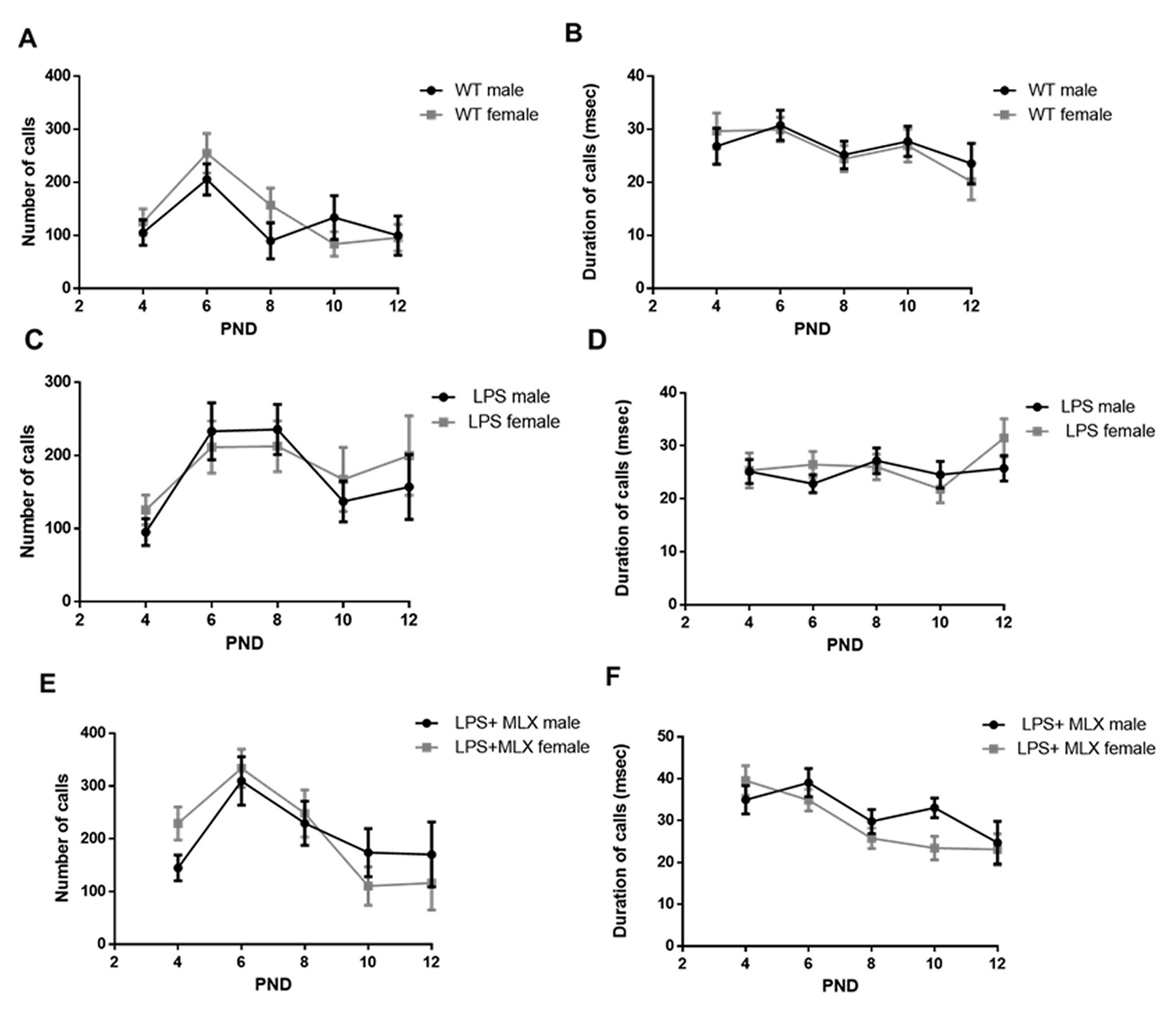

Supplement: Supplementary file 1 [file brainsci-10-00620-s001.zip › suppl figures/suppl figures/Figure S1.tif]

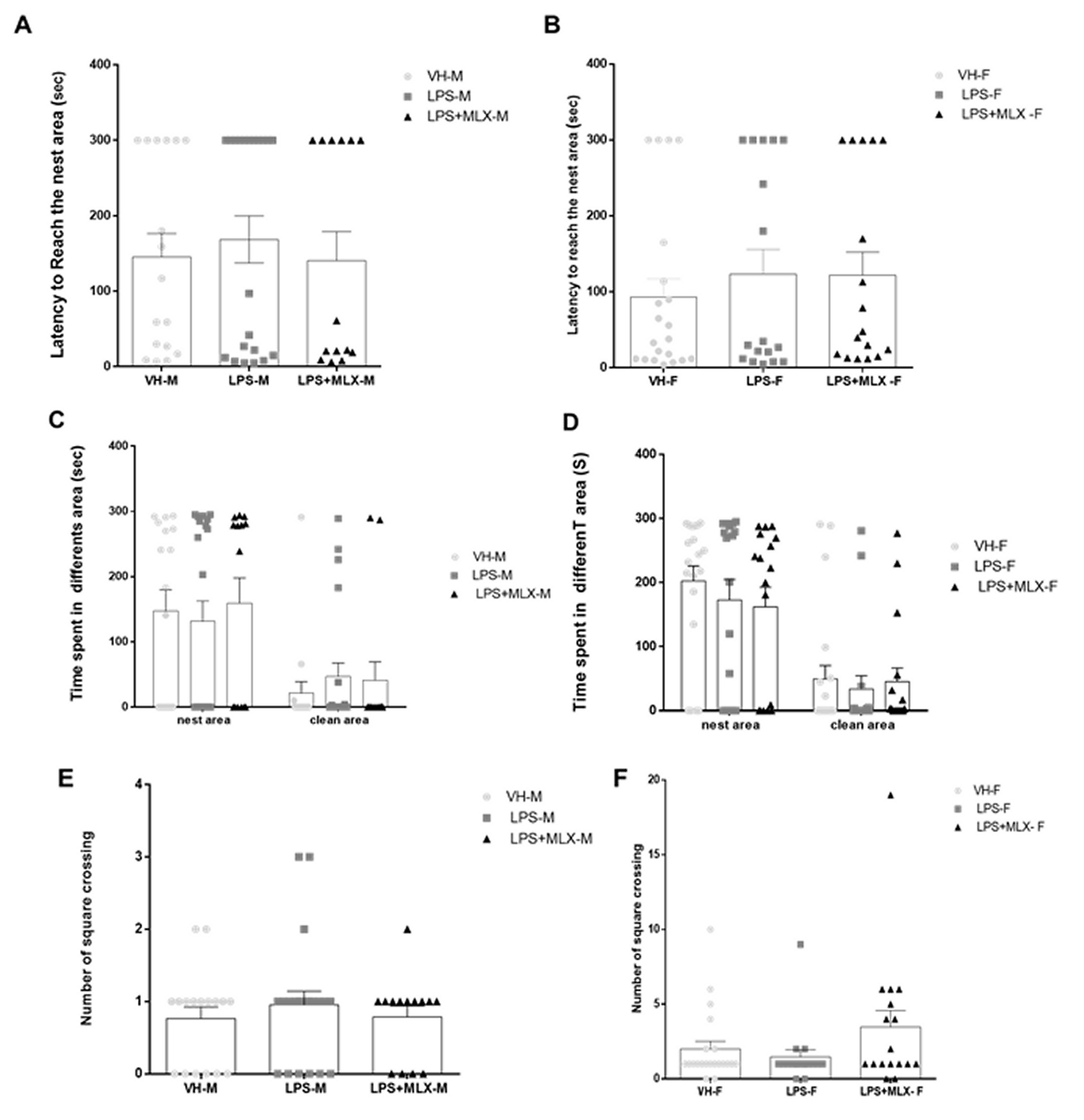

Supplement: Supplementary file 1 [file brainsci-10-00620-s001.zip › suppl figures/suppl figures/Figure S2.tif]

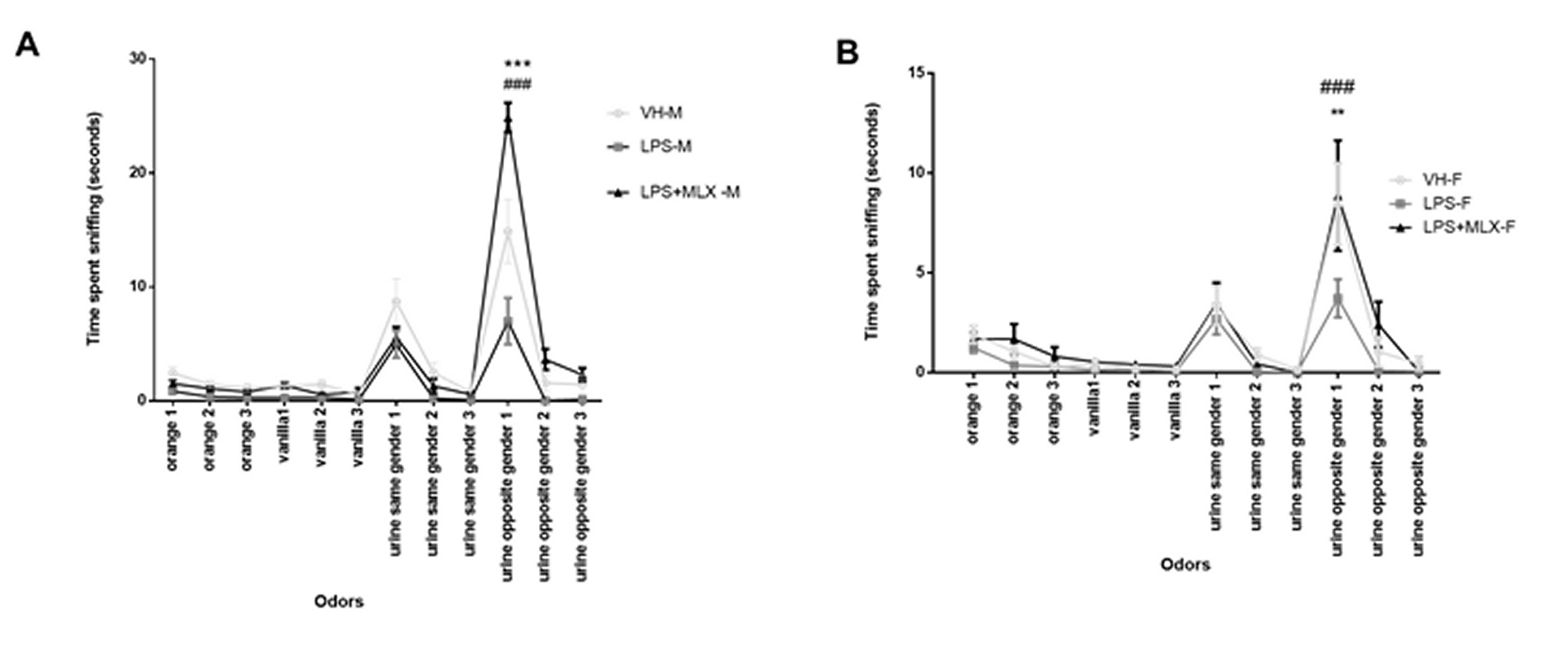

Supplement: Supplementary file 1 [file brainsci-10-00620-s001.zip › suppl figures/suppl figures/Figure S3.tif]

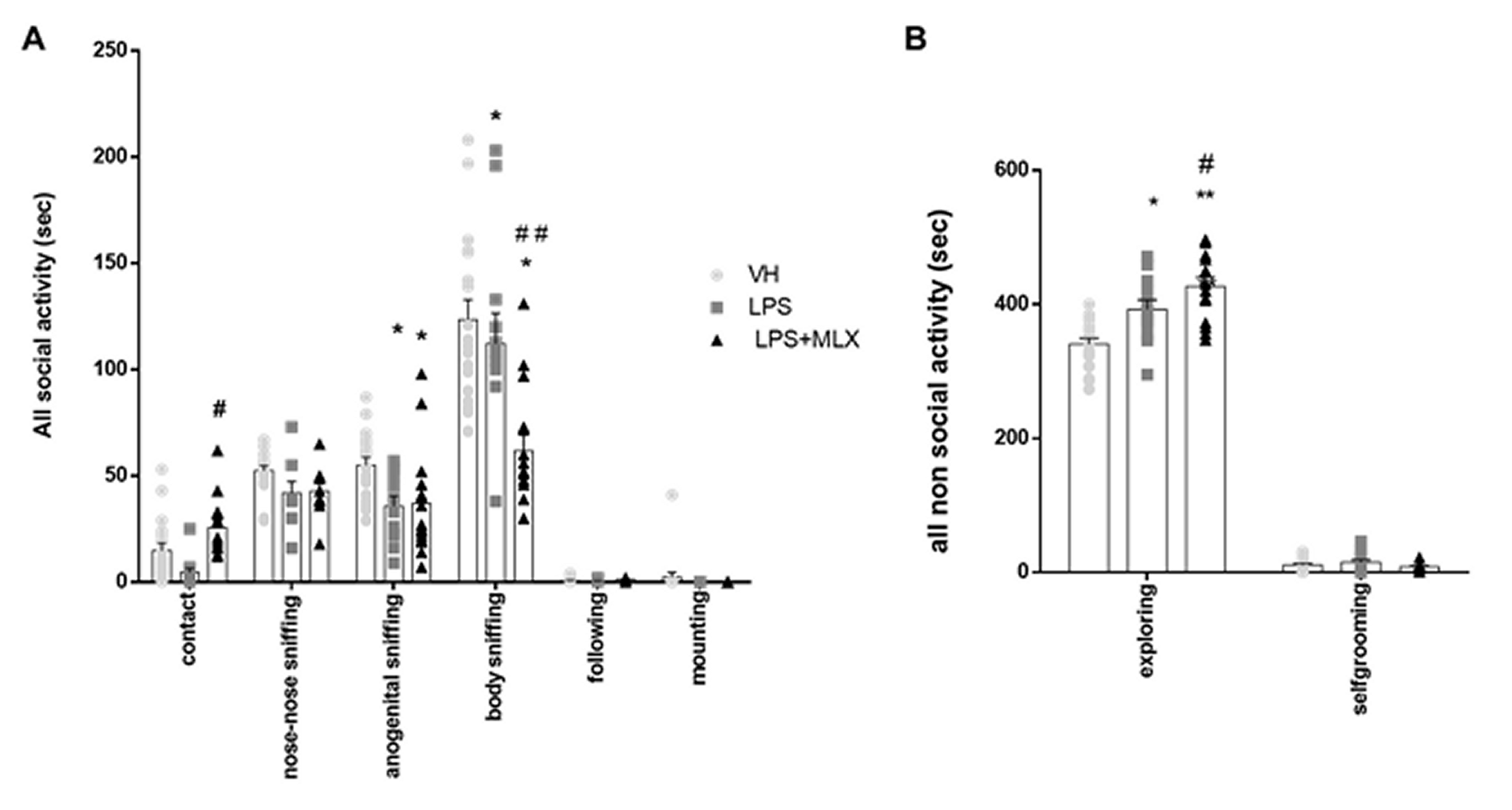

Supplement: Supplementary file 1 [file brainsci-10-00620-s001.zip › suppl figures/suppl figures/Figure S4.tif]
